# Simpson-Golabi-Behmel syndrome types I and II

**DOI:** 10.1186/s13023-014-0138-0

**Published:** 2014-09-20

**Authors:** Jair Tenorio, Pedro Arias, Víctor Martínez-Glez, Fernando Santos, Sixto García-Miñaur, Julián Nevado, Pablo Lapunzina

**Affiliations:** CIBERER, Centro de Investigación Biomédica en Red de Enfermedades Raras, ISCIII, Madrid, 28029 Spain; Molecular Endocrinology – Overgrowth Syndromes Laboratory, INGEMM, Instituto de Genética Médica y Molecular, IdiPAZ, Hospital Universitario la Paz, Universidad Autónoma de Madrid (UAM), Madrid, 28046 Spain; Structural and Functional Genomics - INGEMM, Instituto de Genética Médica y Molecular, IdiPAZ, Hospital Universitario la Paz, Universidad Autónoma de Madrid (UAM), Madrid, 28046 Spain; Clinical Genetics - INGEMM, Instituto de Genética Médica y Molecular, IdiPAZ- Instituto de Investigación Sanitaria del Hospital Universitario La Paz- Universidad Autónoma de Madrid- CIBERER- Centro de Investigación Biomédica en Red de Enfermedades Raras, ISCIII, Madrid, 28046 Spain

**Keywords:** Simpson-Golabi-Behmel Syndrome, Overgrowth, GPC3, GPC4, Macrocephaly, Rare disorders, Glypican, Congenital anomalies, X-linked disorder

## Abstract

Simpson-Golabi-Behmel syndrome (SGBS) is a rare overgrowth syndrome clinically characterized by multiple congenital abnormalities, pre/postnatal overgrowth, distinctive craniofacial features, macrocephaly, and organomegaly. Abnormalities of the skeletal system, heart, central nervous system, kidney, and gastrointestinal tract may also be observed. Intellectual disability, early motor milestones and speech delay are sometimes present; however, there are a considerable number of individuals with normal intelligence.

Genomic rearrangements and point mutations involving the glypican-3 gene (*GPC3*) at Xq26 have been shown to be associated with SGBS. Occasionally, these rearrangements also include the glypican-4 gene (*GPC4*). Glypicans are heparan sulfate proteoglycans which have a role in the control of cell growth and cell division.

Although a lethal and infrequent form (also known as SGBS type II) has been described, only the classical form of SGBS is reviewed in this work, whereas only some specific features on SGBS type II are commented.

We review all clinical and molecular aspects of this rare disorder, updating many topics and suggest a follow-up scheme for geneticists and primary care clinicians.

## Resumen

El Síndrome de Simpson-Golabi-Behmel (SSGB) es un síndrome de sobrecrecimiento raro, que se caracteriza clínicamente por múltiples anomalías congénitas, sobrecrecimiento pre y post natal, rasgos craneofaciales distintivos, macrocefalia y organomegalia. Otras características que pueden presentar estos pacientes incluyen anomalías en el aparato esquelético, el corazón, el sistema nervioso central, el riñón y el tracto gastrointestinal. También pueden presentar discapacidad intelectual, retraso motor precoz y retraso en el habla, aunque en su mayoría, estos individuos presentan una inteligencia dentro de los límites normales.

Los reordenamientos genómicos y las mutaciones puntuales que incluyen el gen *GPC3* (“Glypican-3 gene”) localizado en la región cromosómica Xq26, se han asociado con la aparición del SSGB. Ocasionalmente, estos reordenamientos genómicos pueden incluir el gen *GPC4*. Los glipicanos son proteoglicanos de heparán sulfato que actúan controlando el crecimiento y división celular.

Aunque se ha descrito una forma letal de este síndrome (denominada SSGB tipo II) en esta revisión sólo analizamos la forma clásica de este síndrome y sólo comentaremos algunos aspectos del SSGB tipo II.

En este trabajo se presenta una revisión de todos los aspectos clínicos y moleculares de este síndrome, actualizando algunos aspectos y además se sugiere un esquema de seguimiento de estos pacientes por parte de genetistas y médicos de atención primaria.

## Introduction

The Simpson-Golabi-Behmel syndrome (SGBS) (OMIM 312870;ORPHA373) is an overgrowth/multiple congenital anomalies syndrome caused by mutations in a semi-dominant X-linked gene encoding Glypican 3 (*GPC3*). It shows high clinical variability (Table 1), ranging from very mild forms in carrier females to lethal forms with failure to thrive in males. The most consistent findings in SGBS are pre- and postnatal overgrowth, characteristic facial anomalies and abnormalities affecting the internal organs, skeleton, and in some occasions, variable degree of intellectual disability. SGBS is also associated with an increased risk of developing embryonal tumors (Table 1), mostly Wilms and liver tumors. About 250 patients have been reported so far in the medical literature. The prevalence of the syndrome is unknown.

**Table 1:** **Clinical findings in Simpson-Golabi-Behmel Syndrome Type I (SGBS Type I)**

**1. Craniofacial features**	
Skull	Macrocephaly in 70% of children. Craniosynostosis is observed in many of the cases
Face	Square and coarse. Large forehead. Cleft lip or palate in 13% of the patients. Wide and middle groove tongue
Nose and lips	large and thick
Palate	Cleft palate is present in about 13% of the patients
Tongue	Wide and middle groove from the tip to the back
**2. Tumors**	
Tumor risk	Wilms tumor, hepatoblastoma, adrenal neuroblastoma, gonadoblastoma and hepatocellular carcinoma has been described in patients with SGBS
**3. Cardiovascular abnormalities**	
Cardiac abnormalities	Any type of cardiac abnormalities are very common in SGBS
Cardiovascular malformations	Altered embryonic intracardiac flow
Cardiomyopathy	In about 4% of the cases
Conduction or rhythm abnormalities	In a low percentage of patients
Carotid artery dissection	One case has been described
ECG abnormalities	In about 12% of the patients
**4. Abdominal region**	
Visceromegaly	Nephromegaly, splenomegaly and hepatomegaly is common
Congenital Diaphragmatic Hernia (CDH)	Was observed in less than 10% of the cases reported
Neonatal liver disease	One patient has been described. Finally developed early biliary cirrhosis
Choledochal cyst, biliary cirrhosis	Only reported in 1 patient
**5. Genitalia**	
Hypospadias	These three features have been reported in a low percentage of the patients
Cryptorchidism	
Penoscrotal transposition	
**6. Skeletal**	
Index finger	Hypoplasia
Proximal phalanx	Congenital abnormality
Syndactyly	2nd-3rd finger
Rib malformations	Frequent
Sella turcica	Deep V-shaped
Six lumbar vertebrae	Exceptional
**7. Central Nervous System (CNS)**	
Hydrocephalus	
Epilepsy	
Obstructive sleep apnea syndrome	
Attention deficit hyperactivity disorder	
**8. Speech and language**	
Distorted articulation	Distorted resonance has been also described
Fluency failures	
Stereotype prosody	
**9. Other**	
Neck	Laryngeal web and airway swallow

To date, two different clinical subtypes of SGBS have been described. The classical SBGS (also known as SGBS type I) [1-4] and a lethal and infrequent form (probably with less than 10 cases described; also known as SGBS type II. OMIM 300209; ORPHA79022) described by Terespolsky et al., in 1995 [5,6].

In this work we review all aspects of the classical form of SGBS. Due to the scant number of patients reported with SGBS type II, only some minimal aspects of this disorder are commented in this manuscript.

### Disease definition

SGBS was initially described by Joe Leigh Simpson and coworkers in 1975 [1]. In 1984, Golabi and Rosen [3] and Behmel et al. [2] independently reported further cases. Neri et al. [4] described additional cases, put all these reports together and named the disorder “Simpson-Golabi-Behmel syndrome”. The old terms “Golabi-Rosen syndrome”, “Gigantism-dysplasia syndrome”, “Encephalo-tropho-schisis syndrome” and “Simpson dysmorphia syndrome” and the pejorative “Bulldog syndrome” are no longer in use for SGBS and should be avoided.

### Epidemiology

SGBS syndrome is a rare overgrowth disorder, less common than the Beckwith-Wiedemann and Sotos syndromes. The birth prevalence is unknown. Approximately 250 cases are known to date.

### Clinical description

There are at least two different clinical subtypes of SGBS. The classical form (SGBS or SGBS type I) associated to mutations in *GPC3* and a lethal form (SGBS type II) associated to a different region of chromosome X (Xp22.2) [5]. The lethal form is an infantile lethal variant of SGBS usually associated with hydrops fetalis.

### Clinical findings

#### Clinical subtypes of SGBS

There are two different clinical subtypes of SGBS. The classical SBGS (also known as SGBS type I) [1-4] and a lethal and very infrequent form (known as SGBS type II. OMIM 300209; ORPHA79022) described by Terespolsky et al., in 1995 [5,6]. Clinical findings below correspond mainly to those features observed in classical SGBS type I. Due to the phenomenon of Lyonization; some female carriers may have mild physical findings of SGBS. Some carrier women are tall, have extranumerary nipples, coarse face, abnormal hands and midline defects.

### Skull

Macrocephaly is observed in about 70% of children with SGBS. Craniosynostosis has been reported in many cases [7,8].

### Face

**Figure 1:**
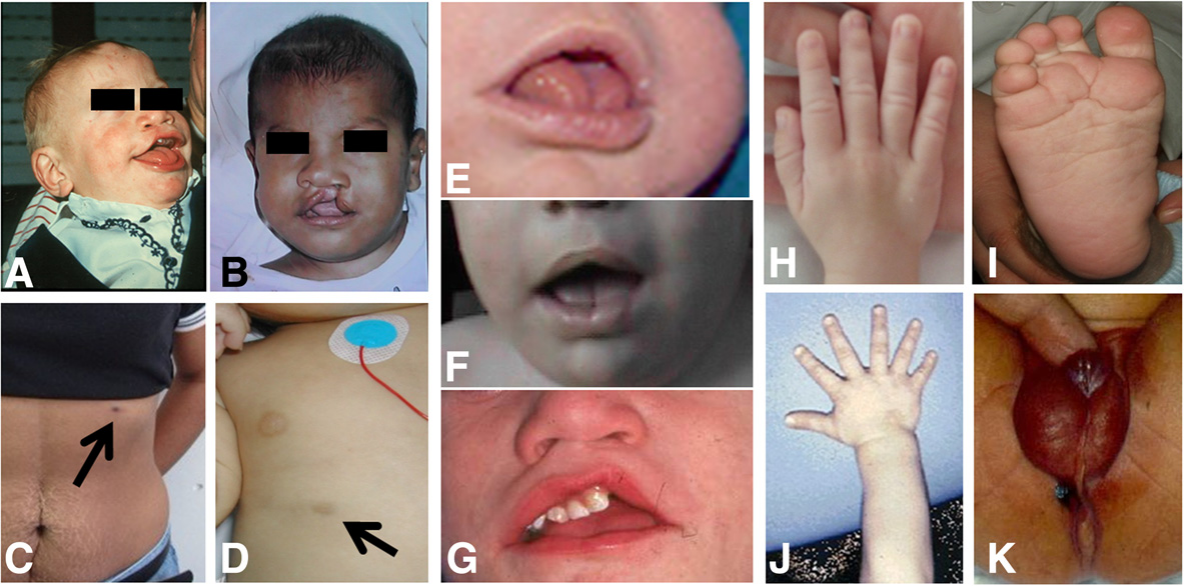
**Clinical findings in Simpson-Golabi-Behmel syndrome. A** and **B**: Facial phenotype. Note the cleft lip, coarse, square face and broad nose. **C** and **D**: extra-nipple in a carrier mother and a toddler. **E**, **F** and **G**: close up of the mouth of three different patients. Note the large tongue, middle groove in the tongue, teeth malposition and repaired cleft (in **G**). **H** and **J**: Hands. Note broad hands and polydactyly in one subject. **I**: Deep plantar creases. **K**: abnormal genitalia in a male with hypospadias and proximal anal placement. Written informed consent was obtained from the parents of the patients for publication of their photographs.

The face in patients with SGBS is square and coarse. The forehead is large and the nose and lips are usually large and thick. Cleft lip and/or palate is observed in about 13% of cases. The tongue is wide and with a middle groove from the tip to the back of the tongue [9-11] (Figure 1). There may be midline minor anomalies such as subcutaneous lipomas, pits or flat nevus flammeus. Multiple odontogenic keratocysts were reported in one patient [12].

### Neck

Laryngeal web [13] and airway and swallow manifestations were reported in a minority of children [14].

### Heart

Diverse cardiovascular malformations have been reported in patients with SGBS, and it is suspected that they are related to the apparently high incidence of early death [15]. Lin et al. [15] reviewed 101 SGBS patients and demonstrated that 36% had a cardiac abnormality, of which 26 had a cardiovascular malformation. Most cases (77%) were class II CVMs (attributed to altered embryonic intracardiac flow). Other cardiac abnormalities included cardiomyopathy (n = 4) and electrocardiogram (ECG) conduction or rhythm abnormalities. Death was associated with a cardiac abnormality in 23% of patients. The authors conclude that cardiac abnormalities of any type are common in SGBS [15].

### Vascular findings

Carotid artery dissection in an adult [16], hepatic vascular malformations [17] and diffuse neonatal hemangiomatosis has been associated with SGBS [18].

### Arrhythmias

Data are insufficient to define a cardiac phenotype/molecular correlation [15]. Lin et al. [15] reviewed 101 cases and reported that 12% of cases had ECG abnormalities, 25% of whom had an underlying cardiovascular malformation. Out of the 29 deaths in Lin and coworkers’ review [15], 9 (31%) were associated with a structural cardiac abnormality, and only one was associated with an ECG abnormality (partial right bundle branch block). Although previous reports indicate neonatal mortality in affected males with SGBS to be as high as 50% [4], there is no clear evidence that arrhythmias have the major role in neonatal mortality without the presence of a major cardiac malformation. There are sufficient data to recommend a baseline echocardiogram and ECG in SGBS patients.

### Thorax

Besides cardiovascular anomalies, supernumerary nipples are common in both affected individuals and carrier females (Figure 1).

### Abdominal region

Visceromegaly (nephromegaly, splenomegaly and hepatomegaly) is quite common. Congenital diaphragmatic hernia (CDH) [19] is observed in less than 10% of children. Neonatal liver disease leading to an early biliary cirrhosis has been reported in one individual who finally needed liver transplantation [20]. Choledochal cyst is also reported in one patient [21].

### Genitalia

Hypospadias, cryptorchidism and penoscrotal transposition have been observed in some individuals [22]. In one patient cryptorchidism also associated chordee of the penis (Figure 1).

### Intellectual disability

Intellectual disability may be present in this disorder. Patients must be carefully evaluated because most of them may have normal intelligence instead of the coarse facial features and difficulties in speech.

### Speech and language

Speech was characterized by a distorted articulation, distorted resonance, fluency failures, and stereotype prosody [23].

### Skeletal

Marked index finger hypoplasia and a congenital abnormality of the proximal phalanx [24] and of the same fingernail, and 2nd-3rd finger syndactyly [25] is observed in many patients. Polydactyly (postaxial) may be found in a minority of individuals (Figure 1). Other skeletal findings include rib malformations, deep V-shaped sella turcica and six lumbar vertebrae [26].

### CNS

Generalized hypotonia is one of the main clinical findings in SGBS. Hydrocephalus, epilepsy [27], obstructive sleep apnea syndrome [28] and attention deficit hyperactivity disorder may be present [29].

### Genotype-phenotype correlations

There is no genotype-phenotype correlation. In a study of genotype-phenotype correlations, Mariani et al. [30] determined that all deletions and point mutations occurring in the eight *GPC3* exons result in loss of function with no phenotypic distinctions based on size or position of a deletion or point mutation [31].

### Penetrance

The penetrance is 100%; all males with a *GPC3* mutation have had clinical findings of SGBS. Penetrance in heterozygous females is not known.

### Tumor risks and tumor predisposition

Affected individuals with SGBS are at increased risk for embryonal tumors, including Wilms tumor, hepatoblastoma, adrenal neuroblastoma, gonadoblastoma, and hepatocellular carcinoma. A review of the main tumors (Wilms, gonadoblastoma, neuroblastoma and liver tumors) observed in SGBS patients have been reported in 2005 [32]. Kosaki et al. [33] reported a SGBS patient with hepatoblastoma and a *CTNNB1* somatic mutation (p.Ile35Ser) together with a germline loss-of function mutation in *GPC3*. Since the *CTNNB1* mutation in the tumor tissue represents a driver mutation, these data suggest that mutation in *GPC3* may influence one of the initial steps in tumorigenesis and the progression to hepatoblastoma. Other patients with SGBS and hepatoblastoma [33,34] both at age 9 months, [35] and at 14 months have been also described. Hepatocarcinoma is less common [11]. Metastatic medulloblastoma in an adolescent has been published [36].

### Etiology

#### SGBS type I

After the mapping efforts done for some groups [37] a translocation [38] in a woman with an X-autosome translocation led to the recognition of *GPC3* as the gene responsible of the disease. Most cases follow an X-linked inheritance though some cases are *de novo*. There is at least one family with germinal mosaicism [39]. Deletions, duplications and point mutations of *GPC3* have been reported in patients with SGBS type I. There is only one report of a duplication of *GPC4* [40]; thus, the role of this gene in the pathogenesis of SGBS type I needs further investigations and evidences. No point mutations of *GPC4* were reported so far in individuals with SGBS type I.

### SGBS type II

There are a small number of reports on this rare form of the disease [5,6,41]. It is an infantile lethal variant of SGBS associated with hydrops fetalis. In the first report, the authors reported 4 maternally-related male cousins with a severe variant of SGBS [6]. One of these males was aborted therapeutically at 19 weeks of gestation following the detection of multicystic kidneys on ultrasound. The three live born males were hydropic at birth. They also depicted craniofacial anomalies including macrocephaly; apparently low-set, posteriorly angulated ears; hypertelorism; short, broad nose with anteverted nares; large mouth with thin upper vermilion border; prominent philtrum; high-arched and cleft palate. Other findings were short neck; redundant skin; hypoplastic nails; skeletal defects involving upper and lower limbs; gastrointestinal and genitourinary anomalies, hypotonia and neurologic impairment. All patients died within the first 8 weeks of life. SGBS type II maps to Xp22 and is postulated to be a distinct disorder with overlapping phenotypic features. Budny et al. [41] identified a *CXORF5 (*also called as *OFD1)* mutation in one family with some clinical findings suggestive of SGBS type II but further analysis of 17 patients with clear phenotypic features of SGBS and negative for *GPC3* mutations found no mutations in *CXORF5* [42]. Thus, *CXORF5* is not clearly associated to SGBS type II.

### The gene and proteins in the pathway

Glypicans (GPCs) are a family of proteoglycans that are bound to the cell surface by a glycosylphosphatidylinositol anchor. Six glypicans have been found in the mammalian genome (GPC1 to GPC6). GPCs regulate several signaling pathways [43]. Given the critical role that insulin-like-growth factor II (IGF-II) plays in the regulation of embryonic growth, it was initially proposed that *GPC3* was an inhibitor of IGF-II, and that the overgrowth observed in the SGBS patients was due to an increase in IGF-II signaling caused by the loss of functional GPC3. However, GPC3 does not interact with IGF-II, *GPC3*-null embryos display normal levels of IGF-II [43-45] and the fact that the crossing of the *GPC3*-null mice with various mouse strains that lacked critical components of the IGF signaling pathway did not show any genetic interaction [46] gave a definitive proof that overgrowth in SGBS patients is independent of IGF. Capurro et al. hypothesized that GPC3 acts as an inhibitor of Hedgehog (Hh) signaling in the embryo, and that the overgrowth found in SGBS patients is due, at least in part, to hyperactivation of hedgehog signaling caused by the loss of functional GPC3, which was demonstrated by the finding that hedgehog signaling activity is elevated in *GPC3*-null mice [47]. The binding of Hh to GPC3 triggers the endocytosis and degradation of the GPC3/Hh complex [47]. Additional evidence that GPC3 is a negative regulator of Hhsignaling was recently provided by some experiments performed in cultured Drosophila cells [43,48].

### Diagnosis

#### Diagnostic criteria

The major diagnostic criteria are: overgrowth (macrosomia, macrocephaly and/or pre-and postnatal overgrowth), coarse, characteristic facial appearance, midline defects and tumor predisposition (Table 1). Other findings are organomegaly, anomalies of the skeletal system, and congenital malformations of the heart, central nervous system, kidney, and gastrointestinal tract. Intellectual disability of variable degree may be present.

### Diagnostic methods

Diagnosis is suspected on the clinical findings, pedigree analysis and medical problems of patients. Genetic approach currently includes a karyotype focusing on the X chromosome, array CGH and/or MLPA analysis and mutation analysis of the *GPC3/GPC4*. Next generation sequencing technologies have allowed the simultaneous testing of many genes responsible of overgrowth through panels of genes or directly through whole exome sequencing [49].

### Laboratory and medical imaging findings

No biochemical or endocrinological pathognomonic markers have been documented in patients with SGBS. Central nervous system findings on CT scan or brain MRI are common; midline defect such as abnormal corpus callosum, central lipomata, and hydrocephalus may be present. On X-rays are very useful a typical sign of index finger hypoplasia and a congenital abnormality of the proximal phalanx [24] and of the same fingernail. Rib malformations are usually observed in most patients.

### Differential diagnosis

SGBS belongs to a group of overgrowth syndromes that have some clinical features in common such as pre- and/or postnatal overgrowth and in some of them cancer predisposition. SGBS syndrome has clinical overlap with other overgrowth syndromes, in particular with Beckwith-Wiedemann syndrome. Beckwith-Wiedemann syndrome due to *CDKN1C* mutations demonstrates the highest clinical similarities with SGBS, including genitourinary malformations, an increased incidence of embryonic tumors, macrosomia, macroglossia, coarse face and ear anomalies. However, the mode of inheritance of SGBS may help to differentiate these disorders. Other entities that should be considered in the general differential diagnosis of SGBS syndrome are: Weaver syndrome, Perlman syndrome; Fragile X syndrome; Bannayan-Zonana syndrome; PTEN hamartoma tumor syndrome; Marshall syndrome, Nevo syndrome; Neurofibromatosis type I; Marfan syndrome; nevoid basal cell carcinoma syndrome (Gorlin syndrome), Fryns syndrome, Elejalde syndrome (acrocephalopolydactylous dysplasia), mosaic trisomy 8, Pallister-Killian syndrome and trisomy 15q26-qter [50].Many of these syndromes can be easily excluded on the base of other major clinical features, pedigree analysis and mode of inheritance.

### Genetic counseling

#### Parents

SGBS is inherited in an X-linked fashion. If there is only one affected individual in a family, the patient may have inherited the mutation from his mother or may have a *de novo* mutation. Reviewing all published cases of SGBS indicates that the frequency of *de novo* mutations is about 20-30%. In families with more than one affected individual, the mother of an affected male may be a carrier (most probably) or may have germline mosaicism (rarely). Chromosome analysis (including FISH) and aCGH/MLPA of the mother needs to be performed when a deletion of *GPC3* is detected in the affected child. Molecular tests looking for point mutations through sequencing methods (Sanger, NGS, etc.) should be warranted for every mother of molecularly-confirmed individual. Germline mosaicism is extremely rare and has been reported in only one family [39].

### Sibs

The risks of brothers and sisters of a patient will depend on the status of the mother. If the mother of a patient is carrier of the molecular defect, the probability of transmitting the disease in each pregnancy is 50%. Brothers who inherit the mutation will be affected by the disorder and sisters who inherit the mutation will be carriers and will usually not be affected or show minimal clinical findings of SGBS [50].

### Offspring

Male SGBS patients will transmit the *GPC3* mutation to all of their daughters and none of their sons that will not be affected by the disease. Daughters of affected male patients will be carriers.

### Antenatal diagnosis and preimplantational genetic testing

#### Ultrasound examination

Ultrasound approach is possible in families at risk of SGBS [50-52]. Increased nuchal translucency and other ultrasound findings such as macrosomia, cleft lip or palate, nephromegaly, macroglossia and hydrops/ascites may suggest the fetus is affected [7,53]. The finding of disproportionate fetal overgrowth together with elevated maternal serum alpha-fetoprotein is also useful to suspect SGBS [19].

### Molecular tests

Once the mutation has been identified in a patient or any family member, prenatal diagnosis and/or preimplantational genetic testing is possible for at-risk pregnancies [50].

### Management & treatment

#### General management

It includes treatment of neonatal hypoglycemia and multidisciplinary support of many pediatric specialists such as cardiologist, neurologist, and orthopedist. Specific management and follow-up of tumors should be warranted to all individuals with SGBS (see below).

### Management for males with SGBS

- Monitoring for hypoglycemia in the newborn period.
- Physical examination to monitor for scoliosis during period of rapid growth rate; radiographs as needed.
- If development appears to be normal on initial assessment, routine monitoring of social and intellectual development.
- Monitoring of renal function if renal anomalies are present.
- Physical examinations to monitor for tumor risk [32]: a) Every three months until age four years; b) Every four months from age four to seven years; c) Biannually after seven years of age.

Main tumors to be screened are: 1) **Wilms and liver tumors:** Abdominal ultrasound examination every three and four months from birth until at least age seven and yearly there after [32,54]. Abdominal ultrasound examination should assess for both Wilms tumor and hepatic tumors. Similarly to Beckwith-Wiedemann syndrome, the risk for Wilms tumor seems to decrease after eight years of age; 2) **Gonadoblastoma.** Serial measurements of serum alpha fetoprotein and beta human chorionic gonadotropin concentrations (also for hepatoblastoma) is recommended [32]; 3) **Neuroblastoma.** Measurements of urinary catecholamine metabolites including vanillylmandelic acid and homovanillic acid as well as urinary free fractionated catecholamines [32] and annual lifelong chest radiograms have also been suggested for chest tumors [32].

### Prognosis

The spectrum of signs and symptoms associated to SGBS is broad, varying from very mild forms in carrier females to infantile lethal forms in affected males. A percentage of affected males die in the newborn period, some of them probably due to heart defects. Carrier females and people with milder cases often live into adulthood. Because of the varying degrees of manifestations and severity associated with the condition, prediction of prognosis and life expectancy most likely varies on an individual basis.

Intellectual disability must be carefully evaluated due to the majority of patients have normal intelligence, and do not have the coarse facial and difficulties in speech as we expected for classical SGBS.

## Abbreviations

CDH: Congenital diaphragmatic hernia
CDKN1C: Cyclin-dependent kinase inhibitor 1C
CNS: Central nervous system
CT: Computed tomography
CTNNB1: Catenin (Cadherin-Associated Protein) Beta 1
CVMs: Cardiovascular malformations
CXORF5 (OFD1): Oral-facial-digital syndrome 1
ECG: Electrocardiogram
EEG: Electroencephalography
FISH: Fluorescence in situ hybridization
GPC3: Glypican 3
GPC4: Glypican 4
Hhsignaling: Hedgehog signaling
IGF: Insulin-like growth factor
IGF-II: Insulin-like growth factor 2
MLPA: Multiplex ligation probe amplification
MRI: Magnetic resonance imaging
NGS: Next generation sequencing
PTEN: Phosphatase and Tensin
SGBS: Simpson-golabi-behmel syndrome
